# Fast and Efficient Separation of Eleven Mycosporine-like Amino Acids by UHPLC-DAD and Their Quantification in Diverse Red Algae

**DOI:** 10.3390/md20060395

**Published:** 2022-06-15

**Authors:** Michael Zwerger, Markus Ganzera

**Affiliations:** Department of Pharmacognosy, Institute of Pharmacy, University of Innsbruck, A-6020 Innsbruck, Austria; michael.j.zwerger@uibk.ac.at

**Keywords:** mycosporine-like amino acids, MAA, isolation, UHPLC

## Abstract

Due to their hostile habitats, characterized by a high exposure to UV-A and UV-B radiation, red algae are known to synthesize unique secondary metabolites: mycosporine-like amino acids (MAAs). These small molecules possess an extremely high UV absorption capacity and therefore mainly act as photoprotective agents. In this study, the first ultrahigh-performance liquid chromatography (UHPLC) method with diode-array detection (DAD) was developed for the determination of eleven MAAs in various algal species. All of the analytes could be separated in under 8 min on a Phenomenex Luna Omega C18 1.6 µm column. The mobile phase comprised water with 0.25% formic acid and 20 mM ammonium formate (A) and acetonitrile (B). Elution was carried out in gradient mode. Method validation following ICH guidelines confirmed excellent linearity (R^2^ ≥ 0.9998), selectivity, precision and accuracy (from 97.41 to 103.38%) for all analytes. The assay’s LOD was always 0.01 µg/mL; its LOQ was not higher than 0.04 µg/mL. Practical applicability was assured by analyzing several algae (e.g., *Gracilaria chilensis*, *Pyropia plicata*) using the developed method, and results indicated a high variation in MAA profiles as well as content. Whilst some MAAs were only found in specific samples, shinorine, which was always present, occurred in concentrations from 0.05 to 4.14 mg/g of dried biomass. As UHPLC-MS was also feasible, this method showed high flexibility concerning the detection mode, surpassing established procedures for MAA analysis not only concerning separation efficiency and analysis time.

## 1. Introduction

Since ozone depletion has diminished the filter capacity of the atmosphere, sunlight reaching the earth’s surface has an increasingly harmful effect on organisms, as UV-A (280–315 nm) and UV-B (315–400 nm) are involved in severe damage on a cellular level [1,2,3,4]. This scenario especially impacts marine organisms in shallow water, which have had to develop unique strategies to cope with these ecologic stressors. In algae, these include self-shading via mat formation or migration to deeper water levels, where UV radiation decreases. However, the synthesis of UV-absorbing metabolites is also an option in this respect [1,2].

Mycosporine-like amino acids (MAAs) are small secondary metabolites (usually below 400 Da) of striking importance in this context. They are nitrogen-rich, water-soluble compounds with cyclohexenone or cyclohexenimine scaffolds [1,4,5,6]. In marine macroalgae, shinorine, porphyra-334 and palythine are the most abundant representatives [1,4], yet over 30 MAAs have been identified to date [7]. The highest concentrations and variability have been observed in Rhodophyta; in brown algae, they are missing, and they are only found in low amounts in marine green algae [1]. However, some terrestrial green algae exhibited MAA concentrations very comparable to those of marine red algae [8]; and they have also been confirmed in cyanobacteria [1,5,6]. Their extremely high molar extinction coefficients between 310 to 360 nm [9], together with pronounced antioxidant capacity [10,11], render them ideal for the treatment of diseases induced by reactive oxygen species (ROS) [5,12] and UV radiation [6,13]. Accordingly, they are suitable for sunscreens [14], as indicated by commercial products such as Helioguard^®^ 365, a formulation composed of enriched algal extracts with porphyra-334 and shinorine. For such products, the term cosmeceutical is sometimes used, as they resemble a cosmetic enriched with bioactive components [15,16]. The latter are MAAs, which indicated promising effects on immunostimulation [17], inhibition of collagenase [18] and skin aging [16,19], reduction of UV-induced DNA impairment [6], as well as anti-inflammatory effects [20,21].

The interest in MAAs has been steadily growing since their discovery in 1960 [22], and efficient methods for their qualitative and quantitative determination are required, especially as the literature reports on significant geographic and seasonal variations in the MAA content even within the same species or genus [1,4,23,24,25]. Until now, the analysis of MAAs has been primarily conducted by conventional HPLC using reversed-type stationary phases [1,4,26,27] or HILIC material [2,23]. Additionally, capillary electrophoresis was evaluated in this respect [28]. One UHPLC method for MAA analysis can be found in the literature. However, it was not used for quantitative purposes (and therefore not validated); there is no representative chromatogram shown to confirm separation efficiency, and “only” cyanobacteria were assessed. The use of 4 µm material for separation is not in agreement with the definition of UHPLC either [29]. Only the HPLC methods developed by Orfanoudaki et al. [1,26] cover a large number of MAAs. However, the required analysis time is close to 40 min. It was therefore the aim of this study to further improve the currently available methods by using UHPLC-DAD for the first time, which should enable the quantitative determination of MAAs in algal extracts by a validated assay in much shorter time.

## 2. Results

### 2.1. Sample Preparation

The respective protocol of Orfanoudaki et al. [26] was followed, yet the algae investigated in this study were available in larger quantities, and therefore, 50.0 mg of biomaterial was used for extraction. Furthermore, the procedure was slightly adjusted for differences in injection volume (UHPLC: 1 µL, HPLC: 5 µL), which could be compensated by reducing the amount of solvent added when redissolving the dried extract (UHPLC: 500 µL, HPLC: 1 mL). The applied procedure was found to be well suitable for UHPLC analysis—without an increase in expenditure whilst still being exhaustive (see Section 3.1).

### 2.2. UHPLC Method Development

**Figure 1:**
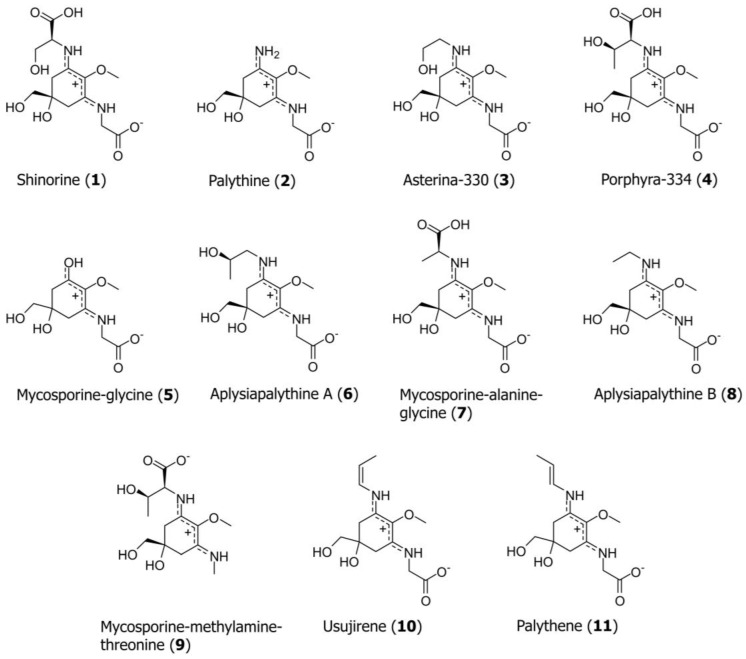
Chemical structure of the MAAs, compounds **1**–**11**, analyzed in this study.

For the development of the UHPLC method, a mixture of eleven MAAs was prepared: shinorine (**1**), palythine (**2**), asterina-330 (**3**), porphyra-334 (**4**), mycosporine-glycine (**5**), aplysiapalythine A (**6**), mycosporine-alanine-glycine (**7**), aplysiapalythine B (**8**), mycosporine-methylamine-threonine (**9**), usujirene (**10**) and palythene (**11**); see Figure 1 for structures. Our investigation started with the search for an optimal stationary phase to permit a fast and efficient separation of all standard compounds. A total of 18 UHPLC columns from different manufacturers, yet all filled with sub-2 µm particles, was evaluated (for an overview of all tested columns, see Supplementary Information, Table S1; chromatograms on selected columns are shown in Figure S1). All standards could (at least partially) be resolved on an Acquity HSS T3 (3.0 mm × 50 mm; 1.8 µm), Waters BEH C18 (2.1 mm × 150 mm; 1.7 µm), Agilent SB C8 (4.6 mm × 50 mm; 1.8 µm) and a Phenomenex Luna Omega C18 100 Å (2.1 mm × 100 mm; particle size 1.6 µm) column; the latter showed the best performance in terms of peak shape and overall separation efficiency, indicated by a peak resolution ≥ 2.04; only in the cases of **10** and **11** was it 1.43. This is not surprising, as both compounds resemble cis/trans-configurational isomers. Thus, the Omega C18 column was selected for further experiments.

Using a mobile phase comprised of pure water as eluent A did not result in satisfying MAA separations because of partial overlap and poor peak shape. In the past, the addition of acids (e.g., acetic acid, formic acid) was described as advantageous, which was the case in the current work also. Whereas adding ammonia was not beneficiary, acidic additives tended to improve the resolution, but some analytes (e.g., **1** and **2**; **4**, **5** and **6**) still coeluted (see Supplementary Figure S2). As several papers reported on the use of formic acid and ammonium formate in combination [1,26,29], this option was evaluated and achieved optimal results. Different concentrations of both additives were assessed, and the best separation was possible by adding 0.25% formic acid and 20 mM ammonium formate to water, which also agrees with previous studies [1,26]. Interestingly, these beneficiary effects could not be observed when using acetic acid and ammonium acetate instead (Supplementary Information, Figure S2).

It quickly became apparent that not all compounds (especially **10** and **11**) could be eluted in a reasonable time in isocratic mode. Therefore, a solvent gradient was required. Besides, to prevent the accumulation of contaminants, a washing step was mandatory. Methanol as solvent B permitted the elution and resolution of compounds **10** and **11**, but this solvent also resulted in a significant increase in column backpressure close to the instrument’s limit of 1000 bar, which was not the case when acetonitrile was selected instead. Our aim was the development of a method providing maximum peak resolution combined with speed. Accordingly, the separation of MAAs was carried out at a flow rate of 0.3 mL/min, which was the optimum value concerning resolution, whereas a part of the re-equilibration step was carried out at 0.4 mL/min, which considerably shortened the required time of analysis. This higher flow rate could not be maintained for the entire equilibration, as some time is required to stabilize the column pressure before the next sample can be injected. When studying the applied gradient, it becomes obvious that **10** and **11** elute when the washing and equilibrating steps have already started. However, considering the dead volume of the column, these analytes still eluted within the gradient, and adverse effects such as distorted peak shapes or the coelution with other compounds in the extracts were not observed. 

**Figure 2:**
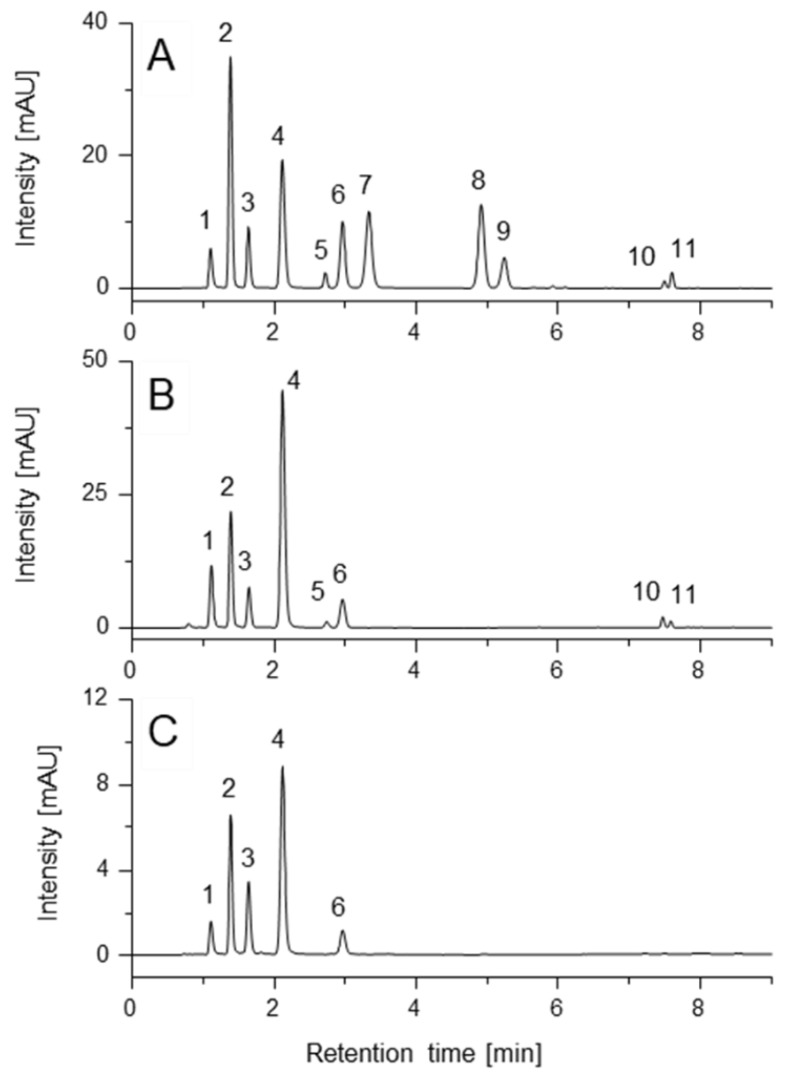
UHPLC-DAD separation of standard compounds (**A**) as well as extracts of *Gracilaria chilensis* (**B**) and *Osmundea* sp. (**C**) under optimized UHPLC-DAD conditions (λ = 330 nm). Stationary phase: Luna Omega C18 100 Å column (2.1 mm × 100 mm; particle size 1.6 µm); mobile phase: water + 0.25% FA + 20 mM ammonium formate (**A**) and ACN (**B**); gradient: in 7 min from 0 to 17.5% B, then switched to 98% B and held for one minute, re-equilibration with initial composition for 5 min; temperature: 15 °C; flow rate during analysis: 0.3 mL/min; sample volume: 1 µL. Compounds: shinorine (**1**), palythine (**2**), asterina-330 (**3**), porphyra-334 (**4**), mycosporine-glycine (**5**), aplysiapalithine A (**6**), mycosporine-alanine-glycine (**7**), aplysiapalythine B (**8**), mycosporine-methylamine-threonine (**9**), usujirene (**10**) and palythene (**11**).

To enhance separation efficiency even further, the effect of column temperature on the separation of MAAs was evaluated. The optimum setting was 15 °C (Figure 2) because at higher temperatures, peaks **6**/**7** and **8**/**9** increasingly coeluted (Supplementary Information, Figure S3). Reducing the temperature below this value did not improve separation; on the contrary, technical problems like water condensation on the column and leak sensors of the instrument increased. The injection volume was set to 1 µL, which is typical for UHPLC and allows for saving of both the sample and standard. The compounds were detected at wavelengths according to the literature (310 nm, 330 nm, 350 nm), which cover the MAA-typical UV absorption range [6,26].

### 2.3. Method Validation

Suitability of the developed method for the quantitative evaluation of mycosporine-like amino acids in real samples, i.e., algal extracts, was confirmed in validation experiments. All respective parameters met the acceptable limits, as can be seen in Table 1, Table 2 and Table 3.

By plotting the peak area against the corresponding standard concentration in the individual calibration solutions, calibration curves in a range from 0.08 µg/mL to 105 µg/mL (or above) could be obtained for **1**–**4**, **6**, **7** and **9**. Calibration data shown in Table 1 highlight the excellent linearity of the method with coefficients of determination (R^2^) always ≥0.9998. Compound **8** was available in very small amount; thus, it was quantified according to the calibration curve of the structurally most similar compound **6**. As stability issues had already been reported for compounds **5**, **10** and **11** [26], they were excluded from quantitative evaluation, although their presence in the samples is indicated in Table 4.

The limit of detection (LOD) was 0.01 µg/mL for all calibrated compounds; the limit of quantification (LOQ) ranged from 0.03 to 0.04 µg/mL. All relevant (i.e., quantified) peaks showed no signs of coeluting impurities in the corresponding DAD or MS spectra. In terms of precision, the developed method exhibited a maximum intraday deviation of 2.77% (corresponding to compound **2** on day 3) and the variance between three consecutive days was not higher than 2.53% (corresponding to compound **6**), as presented in Table 2. Additionally, concerning accuracy, satisfying results were obtained, with recovery rates in the range of 97.41% (corresponding to compound **9**, low spike) to 103.38% (corresponding to compound **1**, medium spike) as presented in Table 3. 

### 2.4. Analysis of Different Algal Species

Several algae, representing different geographical provenances and genera, were analyzed with the UHPLC-DAD method presented here. The results are shown in Table 4. As evidenced there, compound **9** was quantified only in *Pyropia plicata*, whereas **8** could be assigned (but not quantified) just in one *Ceramium* sp. specimen. Compound **1** was present in all algae investigated, and **2** and **4** were present in most of them. However, *Pterocladia* sp. was an exception. As mentioned before, **10** and **11** were excluded from quantitative evaluation, yet they could be detected in *Pyropia plicata* as well as *Gracilaria chilensis* (see respective chromatograms at 350 nm in Supplementary Information, Figure S3). The observed MAA profiles varied qualitatively and quantitatively, for example, reaching from 0.05 mg/g to 4.14 mg/g in case of shinorine, or from 0.03 mg/g to 10.21 mg/g for porphyra-334. Besides, as some samples were already collected between 1995 and 1997 (see supplementary information, Table S2), the stability of the quantified MAAs in dried biomass could be concluded. Seasonal variations, e.g., for *Jania rubens*, were observed as well. However, the number of samples investigated is far too small to draw any chemosystematic conclusions. This was not our intention, because by assaying different algae, “only” the practical applicability of the proposed method should be confirmed.

Additionally, as the optimized mobile phase is volatile, selected extracts were submitted to UHPLC-MS analysis. Corresponding chromatograms are shown as supplementary information (Figure S4). Even when using a different instrument, all MAAs were well separated without changing any of the chromatographic parameters (gradient, temperature, etc.). Retention times only shifted marginally due to different capillary lengths. By operating the MS in positive ESI mode, the identity of all MAAs could be assured in the extracts of *Osmundea* sp. and *Gracilaria chilensis*, thereby confirming that the developed method can be used with an MS a detector as well.

## 3. Materials and Methods

### 3.1. Biomaterial and Preparation of Crude Extracts

Different nori (*Porphyra* sp.) samples were obtained from local supermarkets in Innsbruck 2021; other specimens analyzed (see Section 2.4) were available in-house. They were collected in 2018 in Brittany (France) and morphologically identified by Prof. U. Karsten from the University of Rostock, Germany or had been studied in previous projects already [1]; voucher specimens of all samples are deposited at the Department of Pharmacognosy, University of Innsbruck, Austria.

The dried algal material was finely powdered according to the literature, i.e., frozen with liquid nitrogen and homogenized with a dismembrator (Sartorius Mikro-Dismembrator, Göttingen, Germany). Extraction was achieved by treating 50.0 mg of biomaterial three consecutive times with 5 mL of ultrapure water each time in an ultrasonic bath (Bandelin Sonorex, Berlin, Germany) for 15 min at ambient temperature. The solution was centrifuged (5 min at 1500× *g*), and the clear supernatants were combined and diluted with water to 25.0 mL in a volumetric flask. An aliquot of 1 mL was removed, evaporated under a stream of air and redissolved in 500 µL of water [30]. This enrichment step was crucial to adjust the analyte levels to suitable concentrations for subsequent analysis. As a final step of sample preparation, all extracts were filtered through 0.45 µm syringe filters (Phenex-RC, Ø = 4 mm, cellulose) from Phenomenex (Torrance, CA, USA). Extraction efficiency was assured by extracting one sample (*Osmundea* sp.) a fourth time and analyzing this solution for possibly remaining MAAs. Respective levels were below the limit of detection, so that extraction was considered to be exhaustive.

### 3.2. Chemicals and Reagents

Acetonitrile was purchased from Merck (Darmstadt, Germany), ammonium formate from Serva (Heidelberg, Germany) and formic acid from VWR International (Vienna Austria); all had at least HPLC-grade quality. Ultrapure water was prepared in-house with an Arius purification system (Sartorius, Göttingen, Germany). All solvents and mixtures thereof were filtered through a 0.2 µm membrane filter (Sartorius, Ø = 47 mm, regenerated cellulose) prior to UHPLC use. For the preparation of extracts, ultrapure water was used. Standards were available in-house and were isolated by one of the authors (M.Z.) or during previous projects. Their purity (≥98%) and identity were always confirmed by HPLC-DAD-MS and NMR.

### 3.3. Analytical Conditions

#### 3.3.1. UHPLC-DAD

HPLC analyses were performed on an Agilent 1290-series instrument (Santa Clara, CA, USA) consisting of a quaternary pump, column oven, autosampler and DAD detector. For optimum separations, a Phenomenex Luna Omega C18 100 Å column (2.1 mm × 100 mm; particle size 1.6 µm) protected by a SecurityGuard ULTRA guard C18 pre-column was used. The mobile phase comprised water with 0.25% formic acid and 20 mM ammonium formate (A) and acetonitrile (B). The applied gradient was as follows: from 0 to 17.5% B in 7 min, then immediately changing to 95% B and keeping this composition for one minute (analysis time 8 min); a 5 min re-equilibration step with the initial solvent composition followed. The flow rate during analysis was 0.3 mL/min, 0.4 from minute 8 to 11, and 0.3 mL/min from 11 to 13 min. Column temperature was set at 15 °C and 1 µL of sample was injected. The detection wavelengths were 310 nm, 330 nm and 350 nm, but only 330 nm was used for quantification. 

#### 3.3.2. UHPLC-MS

UHPLC-MS experiments were conducted on an Acquity system from Waters (Milford, MA, USA) comprising a binary pump, autosampler and column oven, which was coupled to a Xevo TQD MS (Waters). The separation conditions were the same as for the Agilent instrument. ESI parameters were set as follows: capillary voltage 3.50 kV, cone voltage 50 V, desolvation gas flow 650 L/h and desolvation gas and source temperature 350 °C and 150 °C, respectively. The MS was operated in positive ionization mode.

### 3.4. Calibration and Method Validation 

The developed UHPLC method was validated according to ICH guidelines [31] to ensure fulfillment of current regulatory standards. Results of the validation are summarized in Section 2.3.

#### 3.4.1. Linearity, Limit of Detection (LOD) and Limit of Quantification (LOQ)

Stock solutions of selected standards (i.e., those available in sufficient amount) were prepared by dissolving the separately weighed substances **1**, **2**, **3**, **4**, **6**, **7** and **9** in 100% water (100 µg/mL). From each of these solutions, not less than 12 calibration levels were obtained by serial dilution 1:1 with water. Each level was measured in triplicate. By plotting the measured peak area against concentrations, calibration curves were generated.

Regression parameters were determined using Microsoft Excel 2019. LOD and LOQ were calculated, whereby the former value was expressed as 3.3 times the standard deviation of y-intercept divided by slope; the limit of quantification corresponded to three times this value [31]. 

#### 3.4.2. Precision and Accuracy

Five individual sample solutions of *Osmundea* sp. were prepared on each of three consecutive days following the above-described extraction protocol. Intraday precision was evaluated based on the results obtained on single days; for interday consistency, the data of all three days were compared. Variations were calculated using the peak areas and expressed as relative standard deviation (RSD). 

Accuracy was assessed by individually spiking the dry sample of *Osmundea* sp. with known amounts of standards at three concentration levels (1, 2.5 and 5 µg/mL, always related to the final sample volume of 25 mL) prior to sample extraction. The respective volumes were added to the accurately weighed biomass placed in a centrifuge tube, and extraction conducted as described in Section 3.1. All solutions were prepared in triplicate, and after analysis, the theoretically present concentration was compared to the actually determined one; the recovery rate was expressed in percent. 

## 4. Conclusions

The method for the determination of eleven mycosporine-like amino acids presented here convinces with excellent separation efficiency in much shorter analysis time than established procedures [1,26]. Compared to previous studies, calibration data now include two additional standard substances (palythine and asterina-330), so the reliability of quantitative results was further enhanced. Three standards had to be excluded from quantification due to stability issues; they were qualitatively assigned, however. The method fulfilled all validation criteria as defined in ICH guidelines and was successfully applied for the analysis of MAAs in different algal species. The obtained results were very similar to those obtained by conventional HPLC. For example, the identical *Osmundea* sp. sample quantified according to [26] yielded the following results in mg/g (UHPLC results in brackets): shinorine 0.09 (0.10), palythine 0.45 (0.50), asterina-330 0.26 (0.28), porphyra-334 0.66 (0.69) and aplysiapalythine A 0.06 (0.07). The method described here utilizes a volatile mobile phase so that the hyphenation to MS was possible, as shown for two examples. This widens its applicability, as—for example—the search for novel MAAs in previously not-studied samples becomes feasible. The use of UHPLC-MS also offers the chance for a further reduction of analysis time, as no chromatographic separation is required anymore; however, due to the widespread use of DAD and the favorable characteristics of MAAs in this respect (i.e., high extinction coefficients), we focused on developing a method using a “classical” detector. Regardless of the detector selected, concerning speed and efficiency, the UHPLC method presented here facilitates MAA analysis on a previously unreached level, hopefully paving the way for many future studies on this highly interesting group of natural products. 

## Figures and Tables

**Table 1:** UHPLC-DAD calibration data for selected compounds **1**–**4**, **6, 7** and **9**, all determined at 330 nm.

Calibration Data					
Compound	Regression Equation	R^2^	Linear Range *	LOD *	LOQ *
**1**	y = 26.408x + 1.6024	0.9999	0.06–124	0.01	0.04
**2**	y = 14.121x + 2.0619	0.9998	0.05–205	0.01	0.03
**3**	y = 18.850x + 2.0478	0.9998	0.05–105	0.01	0.04
**4**	y = 26.120x +2.3516	0.9999	0.06–113	0.01	0.04
**6**	y = 14.598x +2.5545	0.9999	0.08–163	0.01	0.03
**7**	y = 18.789x +2.1740	0.9999	0.06–128	0.01	0.03
**9**	y = 11.766x +1.9540	0.9998	0.08–172	0.01	0.04

* µg/mL.

**Table 2:** Precision of the developed UHPLC method based on peak area. Intra- (*n* = 5) and interday (*n* = 3) variation in percent for sample *Osmundea* sp., extracted and analyzed under optimum conditions. All sample solutions were analyzed in triplicate.

Compound	Relative Standard Deviation			
	Day 1	Day 2	Day 3	Inter-Day
**1**	2.68	2.16	2.62	2.05
**2**	2.67	2.50	2.77	0.71
**3**	1.11	2.33	2.54	2.42
**4**	1.51	1.71	1.61	1.38
**6**	2.12	1.99	1.82	2.53

**Table 3:** Accuracy of the developed UHPLC method. Recovery rates at three different spiking levels for sample *Osmundea* sp., extracted and analyzed under optimum conditions. Three samples per spiking level were prepared, and all solutions analyzed in triplicate; results reflect mean values and relative standard deviations in parentheses.

Compound	Recovery Rate (%)		
	Low	Medium	Low
**1**	101.8 (1.1)	103.4 (1.3)	100.1 (1.2)
**2**	97.5 (0.7)	102.3 (1.1)	102.2 (2.3)
**3**	100.8 (1.1)	102.9 (0.8)	102.8 (2.3)
**4**	98.2 (1.8)	101.6 (1.0)	100.3 (1.5)
**6**	103.2 (1.8)	97.3 (0.6)	103.3 (2.2)
**7**	100.4 (2.6)	102.4 (1.7)	98.8 (1.6)
**9**	101.1 (1.8)	97.9 (1.6)	97.4 (1.9)

**Table 4:** Quantitative results for algae analyzed by the optimized UHPLC-DAD method. Mean values expressed as mg per g of dried biomaterial with corresponding relative standard deviation in parentheses. Assignment of compounds as in Figure 1. Det = Analyte detected but not quantified as below LOQ or instable.

	Compound	1	2	3	4	5	6	7	8	9	10	11
Alga												
*Caloglossa ogasawaraensis*		0.13 (1.57)			3.41 (0.60)	-	-	-	-	-	-	-
*Ceramium* sp. (a)		0.10 (1.02)	0.04 (1.81)	Det	1.31 (0.71)	Det	0.04 (2.02)	Det	Det	Det	-	-
*Ceramium* sp. (b)		1.13 (2.59)	0.54 (1.26)	-	0.06 (1.95)	-	-	-	-	-	-	-
*Chondrus crispus*		0.28 (2.11)	1.36 (1.51)	0.38 (1.48)	-	-	-	-	-	-	-	-
*Euptilorta formosissima*		Det	0.02 (2.47)	Det	Det	-	Det	Det	-	-	-	-
*Gracilaria chilensis*		0.40 (0.47)	1.30 (1.03)	0.35 (0.57)	2.04 (1.17)	Det	0.47 (0.96)	-	-	-	Det	Det
*Gracilaria gracilis*		3.54 (1.29)	-	-	0.03 (2.48)	-	-	-	-	-	-	-
*Grateolupia turuturu*		2.42 (1.81)	1.06 (2.56)	0.04 (1.00)	-	-	-	-	-	-	-	-
*Jania rubens* (a)		0.16 (2.54)	0.08 (1.28)	-	-	-	-	-	-	-	-	-
*Jania rubens* (b)		0.05 (1.92)	0.16 (1.14)	-	-	-	-	-	-	-	-	-
*Mastocarpus stellatus*		2.83 (0.69)	0.29 (1.66)	0.06 (1.51)	0.05 (2.40)	-	-	-	-	-	-	-
*Osmundea* sp.		0.09 (1.07)	0.51 (1.34)	0.27 (1.70)	0.69 (2.03)	-	0.06 (1.28)	-	-	-	-	-
*Porphyra columbina*		1.07 (0.46)	0.11 (0.99)	Det	2.51 (0.65)	0.07 (1.81)	-	0.17 (0.63)	-	-	-	-
*Porphyra* sp. (Nori a)		0.17 (0.82)	0.36 (1.30)	0.45 (1.15)	6.58 (1.17)	-	-	-	-	-	-	-
*Porphyra* sp. (Nori b)		0.21 (0.52)	0.13 (1.34)	0.27 (0.64)	4.36 (1.94)	-	-	-	-	-	-	-
*Porphyra* sp. (Nori c)		4.14 (0.52)	0.17 (2.46)	-	5.62 (1.92)	-	-	-	-	-	-	-
*Pterocladia sp.*		0.20 (0.63)	Det	-	Det	-	-	-	-	-	-	-
*Pyropia plicata (a)*		2.38 (0.40)	1.52 (0.15)	Det	3.82 (0.41)	-	0.05 (1.46)	0.38 (1.12)	-	0.10 (1.92)	-	-
*Pyropia plicata (b)*		1.60 (0.83)	0.77 (1.41)	Det	2.40 (0.51)	Det	0.05 (1.51)	0.11 (0.89)	-	-	Det	Det
*Pyropia plicata* (c)		1.24 (0.50)	0.33 (1.79)	Det	2.13 (0.56)	-	0.03 (1.99)	0.13 (0.40)	-	-	Det	Det
*Pyropia umbilicalis*		0.77 (0.56)	0.76 (1.17)	0.13 (1.69)	10.21 (0.24)	-	0.62 (0.73)	0.11 (1.51)	-	-	-	-
*Schizymenia apoda*		0.04 (0.88)	0.02 (2.81)	-	Det	-	-	-	-	-	-	-
*Spongoclonium pastorale*		0.10 (0.37)	Det	-	3.51 (0.36)	-	-	-	-	-	-	-

## Data Availability

Not applicable.

## Supplementary Materials

The following supporting information can be downloaded at: https://www.mdpi.com/article/10.3390/md20060395/s1, Figure S1: Separation of MAAs on excluded stationary phases; Figure S2: Influence of different mobile phase compositions; Figure S3: Influence of temperature and gradient; Figure S4: UHPLC-chromatogram of *Gracilaria chilensis* at 350 nm; Figure S5: UHPLC-MS analysis of two extracts in SIR; Table S1: Overview of all tested stationary phases; Table S2: Provenance and taxa of the analyzed algae.
