# Supplementary material for: Fast and Efficient Separation of Eleven Mycosporine-like Amino Acids by UHPLC-DAD and Their Quantification in Diverse Red Algae
Source: Mar Drugs. 2022 Jun 15;20(6):395. doi: 10.3390/md20060395 (PMC9227160; doi:10.3390/md20060395)
Supplement: Supplementary file 1 [file marinedrugs-20-00395-s001.zip › marinedrugs-1744378-supplementary.pdf]

Fast and efficient separation of eleven mycosporine-like amino acids by UHPLC-DAD and their quantification in diverse red algae

Supplementary information

Michael Zwerger, Markus Ganzera*

Institute of Pharmacy, Pharmacognosy
University of Innsbruck
Innsbruck, Austria

*Corresponding author:
Assoz. Prof. Dr. Markus Ganzera
Institute of Pharmacy, Pharmacognosy, University of Innsbruck
Innrain 80-82, 6020 Innsbruck, Austria

1. UHPLC method development

Table S1. Overview of all tested stationary phases.

Stationary phase	Dimensions
**Agilent BIO SCX NP**	(4.6 mm x 50 mm; 1.7 µm)
**Agilent SB C8**	(4.6 mm x 50 mm; 1.8 µm)
**Agilent XDB-C18**	(4.6 mm x 50 mm; 1.8 µm)
**Fortis C18**	(2.1 mm x 50 mm; 1.7 µm)
**Grace Vision HT C18 P**	(2.0 mm x 50 mm; 1.5 µm)
**Macherey-Nagel Nucleodur C18 Isis**	(2.0 mm x 75 mm; 1.8 µm)
**Phenomenex Kinetex C18**	(2.1 mm x 50 mm; 1.7 µm)
**Sepax BR-C18**	(2.1 mm x 50 mm; 1.8 µm)
**Sepax GP-C4**	(2.1 mm x 50 mm; 1.8 µm)
**VDS optilab Pronto Pearl NPP**	(3.0 mm x 50 mm; 1.5 µm)
**Waters Acquity BEH 130 C18**	(2.1 mm x 50 mm; 1.7 µm)
**Waters Acquity BEH C18**	(2.1 mm x 50 mm; 1.7 µm)
**Waters Acquity BEH C18**	(2.1 mm x 150 mm; 1.7 µm)
**Waters Acquity BEH C8**	(1.0 mm x 50 mm; 1.7 µm)
**Waters Acquity CSH C18**	(2.1 mm x 50 mm; 1.7 µm)
**Waters Acquity CSH Fluorophenyl**	(2.1 mm x 50 mm; 1.7 µm)
**Waters Acquity HSS T3**	(3.0 mm x 50 mm; 1,8 µm)

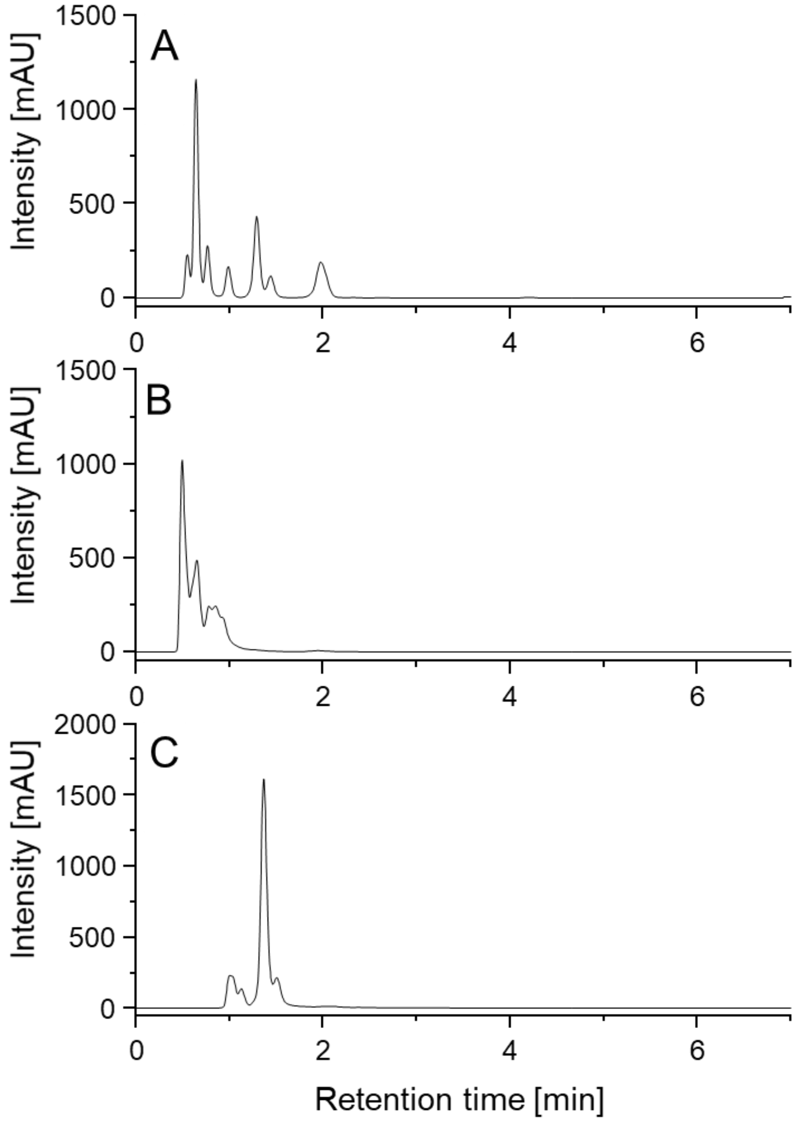

Figure S1. Selection of excluded stationary phases based on the analysis of a standard mix extract (2 mg/ml in water) monitored at 330 nm. (**A**): Fortis C18 (2.1 mm x 50 mm; 1.7 µm); (**B**): Acquity CSH Fluorophenyl (2.1 mm x 50 mm; 1.7 µm), (**C**): Agilent BIO SCX NP (4.6 mm x 50 mm; 1.7 µm). All other conditions were optimal.

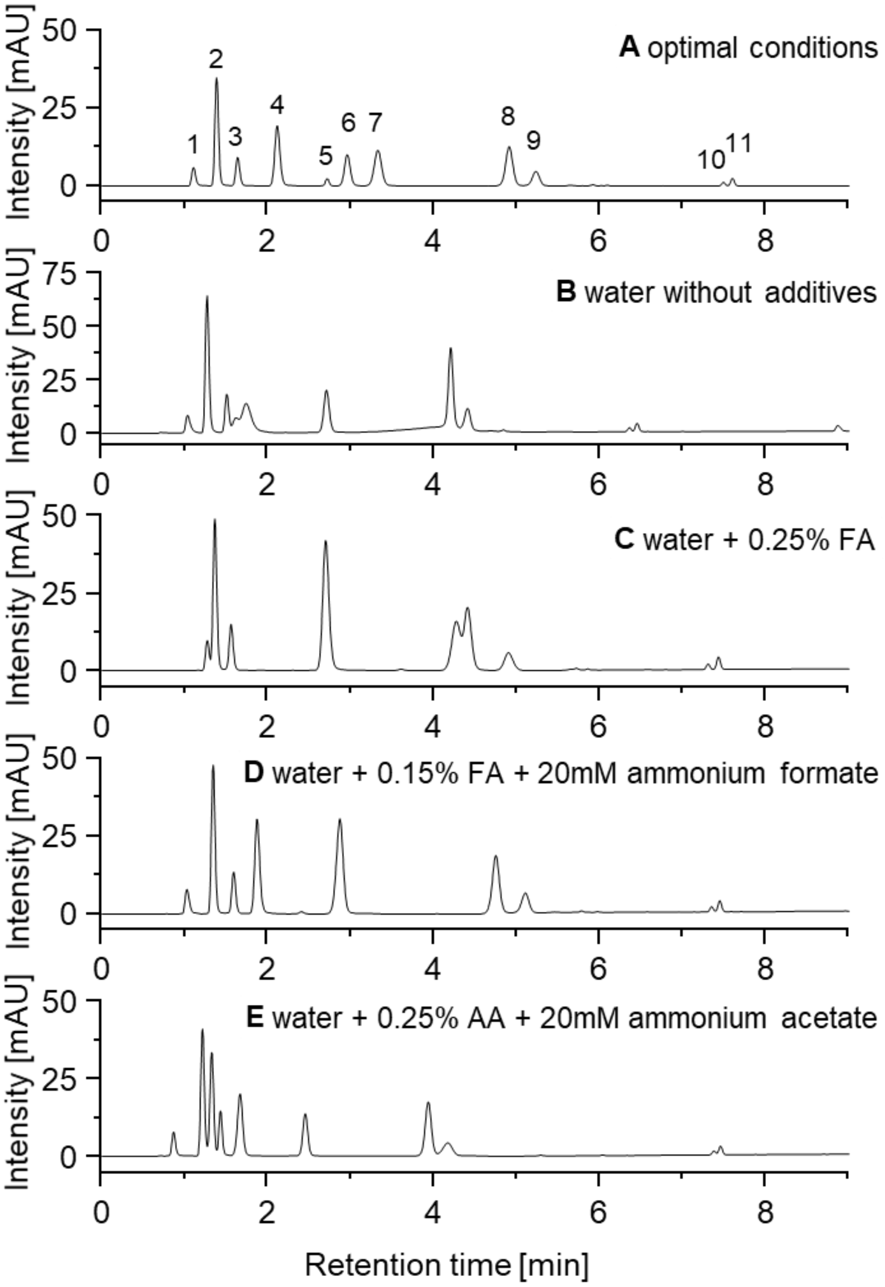

Figure S2. Influence of different mobile phase compositions: Optimum (**A**), water without additives (**B**), water only with 0.25 % formic acid (**C**), water with 0.15 % formic acid and 20mM ammonium formate (**D**), and the result with the addition of 0.25 % acetic acid and 20mM ammonium acetate (**E**). All other settings were optimal.

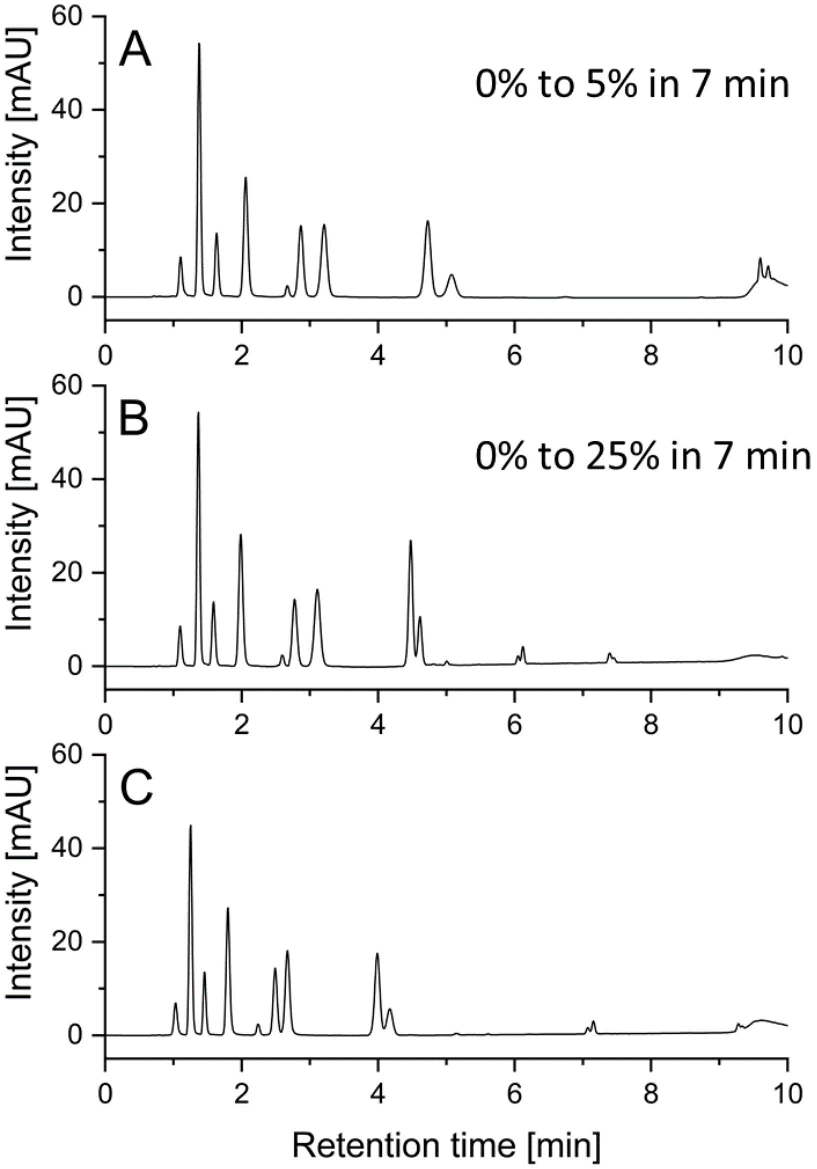

Figure S3. Influence of temperature and gradient on the separation of eleven MAA standards: Gradient too flat and elution of peaks **10** and **11** in the reequilibration step (**A**), gradient too steep and coelution of **8** /**9** and **10**/**11** (**B**), separation at 30°C (**C**). All other settings were optimal.

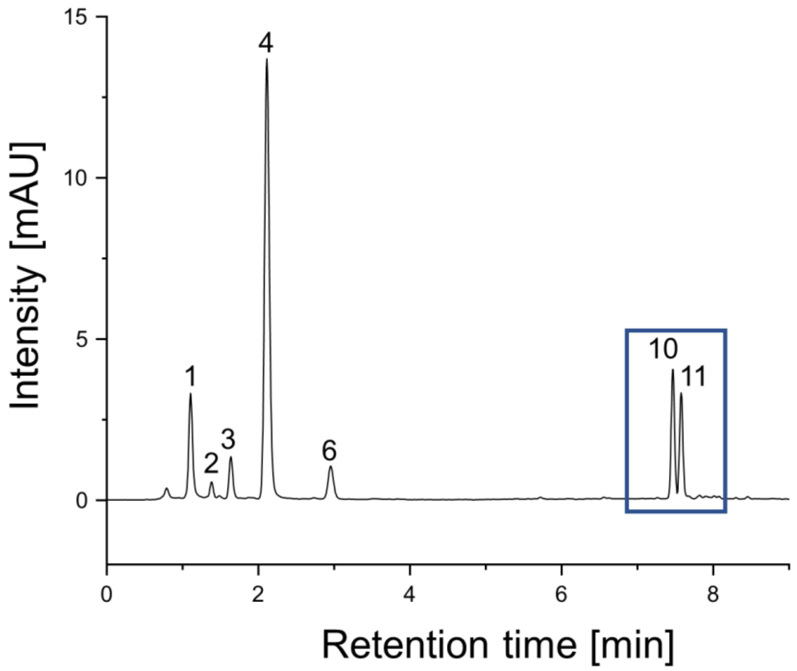

Figure S4. UHPLC-chromatogram of *Gracilaria chilense* at 350 nm under optimized separation conditions for better visibility of compounds **10** and **11**.

2. Analyzed algal samples

Table S2. Provenance and taxa of the analyzed algae

Species	Family, Order	Collection Place	Country	Collection date
Caloglossa ogasawaraensis	Delesseriaceae, Ceramiales	Tokyo	Japan	08/2019
***Ceramium* sp. (a)**	Ceramiaceae, Ceramiales	Wellington	New Zealand	11/2016
***Ceramium* sp. (b)**	Ceramiaceae Ceramiales	Roscoff	France	6/2018
Chondrus crispus	Gigartinaceae, Gigartinales	Roscoff	France	6/2018
Euptilorta formosissima	Callithamniaceae, Ceramiales	Wellington	New Zealand	11/2016
Gracilaria chilensis	Gracilariaceae, Gracilariales	Wellington	New Zealand	2016
Gracilaria gracilis	Gracilariaceae, Gracilariales	Roscoff	France	6/2018
Grateolupia turuturu	Halymeniaceae, Halymeniales	Roscoff	France	6/2018
***Jania rubens* (a)**	Corallinaceae, Corallinales	Roscoff	France	6/2018
***Jania rubens* (b)**	Corallinaceae, Corallinales	Crete	Greece	4/2018
Mastocarpus stellatus	Phyllophoraceae, Gigartinales	Roscoff	France	6/2018
***Osmundea* sp.**	Rhodomelaceae, Ceramiales	Roscoff	France	6/2018
Porphyra columbina	Bangiaceae, Bangiales	Sydney	Australia	1995
***Porphyra* sp. (Nori a)**	Bangiaceae, Bangiales	Not known*	South Korea	Not known*
***Porphyra* sp. (Nori b)**	Bangiaceae, Bangiales	Not known*	Japan	2017
***Porphyra* sp. (Nori c)**	Bangiaceae, Bangiales	Not known*	Spain	Not known*
Pterocladia sp.	Pterocladiaceae, Gelidiales	Wellington	New Zealand	7/2016
Pyropia plicata (a)	Bangiaceae, Bangiales	Wellington	New Zealand	8/2016
Pyropia plicata (b)	Bangiaceae, Bangiales	Wellington	New Zealand	2015
***Pyropia plicata* (c)**	Bangiaceae, Bangiales	Wellington	New Zealand	11/2016
Pyropia umbilicalis	Bangiaceae, Bangiales	Helgoland	Germany	1997
Schizymenia apoda	Schizymeniaceae,Nestomatales	Wellington	New Zealand	2016
Spongoclonium pastorale	Ceramiaceae, Ceramiales	Wellington	New Zealand	11/2016

* commercial sample

3. Mass spectrometry

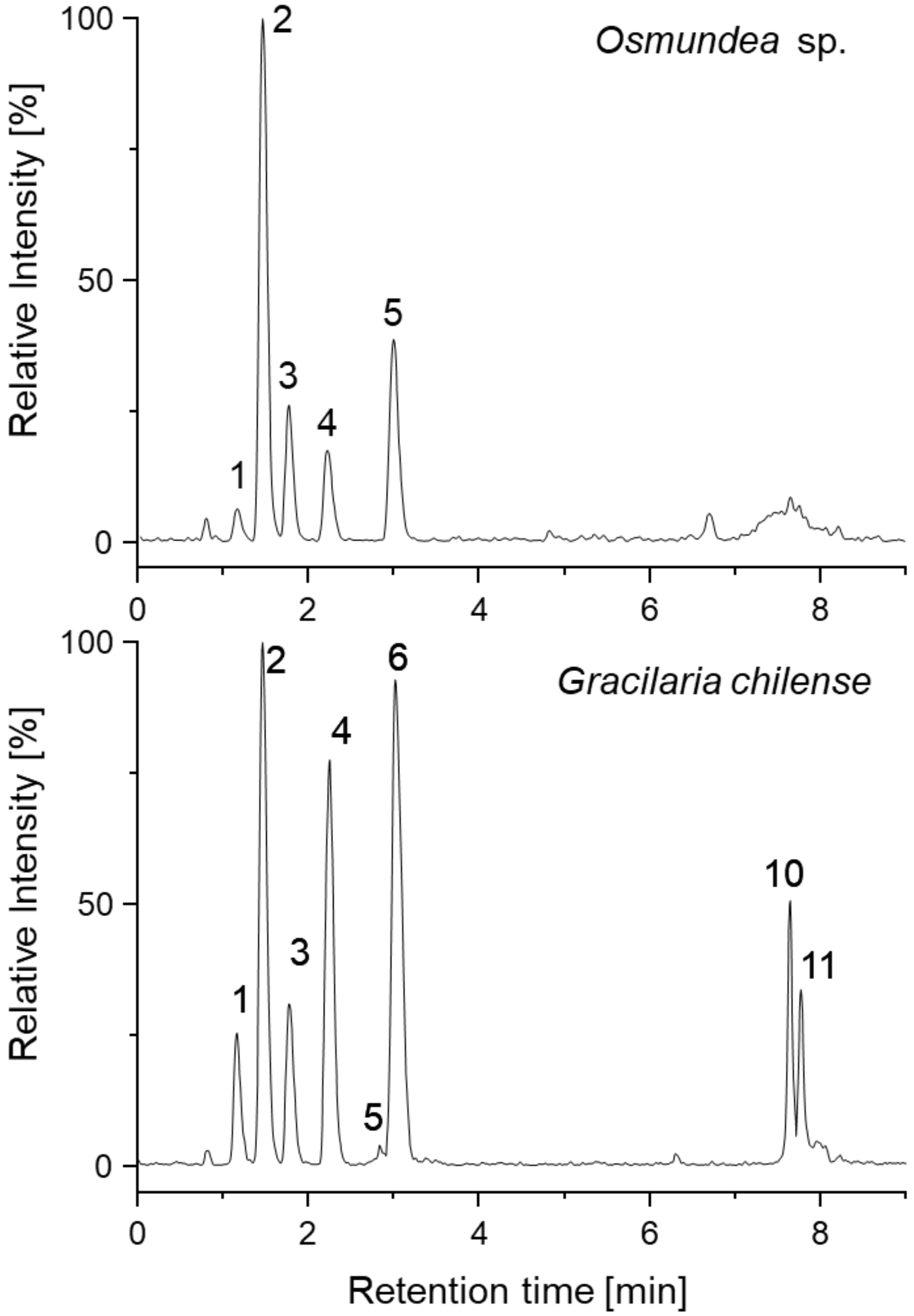

Figure S5. UHPLC-MS analysis of two extracts in SIR (Selected Ion Recording) mode, UV traces of the same extracts are shown in main text. Conditions optimal, i.e. as described in Materials and Methods section.
